# Efficacy and safety of Shenqi Jiangtang Granules plus oral hypoglycemic agent in patients with type 2 diabetes mellitus: A protocol for systematic review and meta-analysis of 15 RCTs: Erratum

**DOI:** 10.1097/MD.0000000000025092

**Published:** 2021-03-05

In the article, “Efficacy and safety of Shenqi Jiangtang Granules plus oral hypoglycemic agent in patients with type 2 diabetes mellitus: A protocol for systematic review and meta-analysis of 15 RCTs”,^[1]^ which appears in Volume 100, Issue 5 of *Medicine*, “a protocol” was included incorrectly in the title.
